# Bacterial Diversity and CAZyme Potential Revealed in Pandanus Rich Thermal Spring Cluster of India: A Non-cultivable 16S rRNA Sequencing Approach

**DOI:** 10.3389/fmicb.2021.760573

**Published:** 2021-11-25

**Authors:** Sangita Dixit, Mahendra Gaur, Enketeswara Subudhi, Rajesh Kumar Sahoo, Suchanda Dey, Lakshmi Datta Mahapatra, Surajit De Mandal, Nachimuthu Senthil Kumar, Hardik Anirudh

**Affiliations:** ^1^Center for Biotechnology, School of Pharmaceutical Sciences, Siksha ‘O’ Anusandhan (Deemed to Be University), Bhubaneswar, India; ^2^Deputy Director Geology, Panchayati Raj and Drinking Water Department (Government of Odisha), Bhubaneswar, India; ^3^Laboratory of Bio-Pesticide Creation and Application of Guangdong Province, College of Agriculture, South China Agricultural University, Guangzhou, China; ^4^Department of Biotechnology, Mizoram University, Aizawl, India; ^5^Department of Electronics and Communication, Dayananda Sagar College of Engineering, Bengaluru, India

**Keywords:** Deulajhari, 16S rRNA, thermal spring cluster, carbohydrate active enzyme, plumbing system, bacterial diversity

## Abstract

In the present study, we explored four different geothermal spots of the Deulajhari spring cluster at a proximity of 10–20 meters with temperatures of 43 to 65°C to unravel their genesis, bacterial diversity and CAZyme potential. However, minor variations in physicochemical properties; TOC, sodium, chloride, zinc and nitrate were observed, including the pH of the spring openings. Illumina based amplicon sequencing revealed *Firmicutes, Proteobacteria* and *Chloroflexi* as the major bacterial phylum with higher abundance in the DJ04 sample. The alpha diversity of all the springs was almost same, whereas beta diversity revealed variations in the degree of uniqueness of OTUs at different temperatures. Statistical analysis established a positive correlation between sulfur content with *Heliobacterium, Thermodesulfovibrio, Thermodesulfobacterium* and *Herpetosipho* as well as TOC and HCO_3_ with *Thermoanaerobacter, Desulfovibrio, Candidatus solibacter* and *Dehalogenimona*. The major hydrocarbon family genes and Carbohydrate Active Enzyme pathways were predicted to be highest in DJ04 with elevated concentrations of HCO_3_ and TOC. Higher homogeneity in geo-physicochemical and microbial features direct the possibility of the common origin of these springs through plumbing systems. However, the minor variations in diversity and functionality were due to variations in temperature in spring openings through the mixing of subsurface water contaminated with carbohydrates from leaf biomass litter. Functional characterization of the thermophilic bacteria of this spring provides essential scope for further industrial applications. The biogeochemical reasons hypothesized for the genesis of unique multiple openings in the cluster are also of interest to conservation scientists for taking measures toward necessary laws and regulations to protect and preserve these springs.

## Introduction

Thermal springs are niches of extreme conditions and are natural laboratories that enable us to comprehend the role of the prevailing environmental features on physiology, diversity, evolution and the functional potential of the inhabiting microbiome. To date, these natural resources have rarely been explored for prospective industrially relevant molecules (Sahoo et al., 2017, 2020). Geothermal manifestations are the outcomes of cumulative natural events of heating water into steam at the geothermal basin and their reappearance on the earth’s surface as an expression of internal energy compounded by the heating of rocks, associated minerals, and gas products. The nature, characters, and modes of different manifestations result in oozing out of the water at the point of fractures in the form of thermal springs, pools, mud pots, geysers, and fumaroles (USGS, 2020). Previous studies have examined the correlation between the unique underground hydrothermal plumbing system and physicochemical features of the water body in the extreme environments underneath these varieties of hydrothermal features (USGS, 2020). In addition, the interaction of local cold surface runoff water, meteoric water, and groundwater and dynamic changes in the subsurface environment due to physical mixing, chemical reaction, and biological activity of microorganisms has been initiated in Caldera and Wyoming, YPN and United States (Christiansen et al., 2007).

In India, around 340 thermal springs are located under ten geothermal provinces, out of which Mahanadi geothermal province in the state of Odisha is the least explored. Lately, the eight thermal springs in this state have gained attention for future geo-exploration endeavors (Chandrasekharam and Chandrasekhar, 2010). The Deulajhari geothermal manifestation cluster with multiple alive water discharge points and a temperature gradient of around 25°C within a proximity of a few hundred meters is situated along the Mahanadi river shear plane under the Eastern Ghats supergroup geological setting. The water released from the multiple spouts is clustered together closely in a single location at Deulajhari, half of which remains cold, even though connected to the neighboring springs with hot water (India TV News Desk, 2013; Mahala, 2019). Taking this unique setting into account, this study aimed to explore (i) whether these spring clusters have a common origin and are interconnected through a plumbing system (ii) if there exist any variations in geothermal and physicochemical properties, and (iii) if the diverse inhabiting microbiome drives the divergence in biological activities. The fascinating feature of this hot spring complex is that it extends over 1.5 acres covered with indigenous luxuriant Pandanus Forest (locally known as Kibana). The hydrocarbon molecules seeping through the thick mat of Pandanus leaf biomass litter in runoff water contaminate the ground water as well as the springs (Lee et al., 2018).

Earlier studies on Deulajhari hot springs are very few and focused either on the geology or undertook the hydro-geochemistry analysis, respectively (Pradhan and Jena, 2016; Mahala, 2019). Other studies have investigated the microbial diversity of the spring (Badhai et al., 2015; Singh and Subudhi, 2016b). One of our preliminary studies tried to understand the microbial diversity from two sampling points (S1 and S2) (Singh and Subudhi, 2016a). However, these findings did not record multiple outflows and failed to explain microbiome dynamics among complex interconnected multiple spring clusters with varying temperature and physicochemical properties. These did not predict the functional activities due to anticipated cellulose contamination of the surrounding dense Pandanus vegetation and their role in reshaping the present community structure. Therefore, these two samples are also included in the present study. The heated water charged with minerals, aromatic compounds and other curative molecules impart therapeutic properties for which Deulajhari has earned the adage “magic water” and became a holy pilgrimage site within local traditions (Deulajhari, 2020; Manisha, 2021). Our recent report on spring water (the main “kund” near the temple) located at the entry point of Deulajhari following the amplicon approach revealed a considerable amount of information on the prevalence of diverse microbial populations and potential biomass-degrading bacteria (Dixit et al., 2021). The main spring opening had a temperature of around 58°C, surrounded by fewer pandanus plants with sediment samples collected in the winter season. We then explored the spring cluster further by enhancing the sample number to four to accommodate the maximum possible temperature gradient and physicochemical parameters.

In the present study, four prominent live samples were subjected to high throughput 16S rRNA amplicon sequencing using the Illumina MiSeq platform to explore the microbial community structure heterogeneity of the Deulajhari thermal cluster. We further attempted to analyze the physicochemical features of the sample to find out the effect of surface runoff water percolating through this dense forest leaf biomass on the Deulajhari springs. The anticipated dynamic changes due to physical mixing, chemical reaction and biological activity of microorganisms in these spouts were predicted in terms of the differential prevalence of metabolic pathways with particular reference to carbohydrate metabolism. Finally, statistical analysis was performed to establish the correlation between the physicochemical features and the functionality of the microbiome of the thermal spring water.

## Materials and Methods

### Sample Collection

**FIGURE 1:**
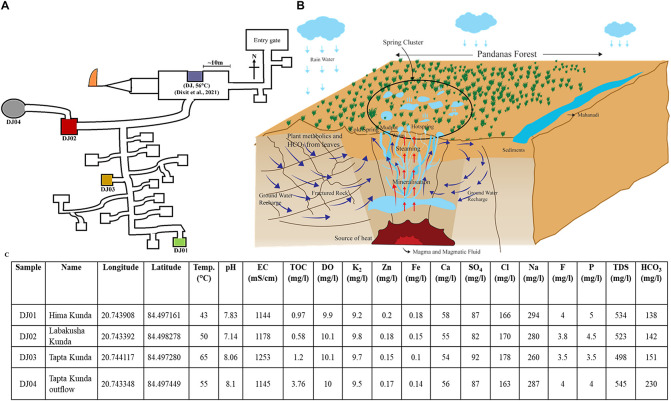
Location of sampling sites, cross-section view of the plumbing system, and physio-chemical features of Deulajhari hot spring cluster. **(A)** Illustrative map of the Deulajhari hot springs cluster. **(B)** The cross-sectional view of the hypothetical underground plumbing system of hot springs to. **(C)** Physio-chemical characteristics, latitude, and longitude of the collected samples from four hot spring points.

The Deulajhari hot spring cluster is positioned at 6 Km northeast of Athamallik in the Angul district, Odisha, India (latitude 20°44′38.997″N and longitude 84°29′49.8624″E). Samples from the four different locations (i.e., Himakunda (DJ01), Labakusakunda (DJ02), Taptakunda (DJ03), and Taptakunda outflow (DJ04) of the thermal spring cluster were collected possessing the highest gradient of the temperature (Figures 1A,B). The distance between the four thermal springs at Deulajhari is approximately 10–20 meters. The thermal spring located at the entry point of the Deulajhari was sampled for microbial diversity study in an earlier study (Dixit et al., 2021), as shown in Figure 1A. Sample collection from these locations (kunds) was performed in January 2016. For each kund, nearly 500 gm of sediment samples were collected using a customized stainless steel funnel attached to a five feet rod and stored into the four different autoclave amber glass bottles (500 ml) with proper labeling as DJ01 (Himakunda), DJ02 (Labakusakunda), DJ03 (Taptakunda), and DJ04 (Taptakunda outflow) instead of water for obtaining better homogeneity (Ferris, 2000; Stolz, 2000). The temperature and pH of each sample were measured *in situ* at the sampling site using an MT-222 Digiflexi digital thermometer (Dr. Morepen, India) and portable pH meter (Hanna Instrument, Sigma, United States) respectively. Sample bottles were kept in dry ice and then transported immediately to the research laboratory and stored at −20°C until further analysis.

### Physiochemical Analysis and DNA Extraction

Nearly 100 gm of sediments from each sample point was centrifuged to separate and collect the sediment. Samples were then filtered through a 5 μm filter pore size (Whatman filter, Merck KGaA, Darmstadt, Germany). Then, the filtered samples were sent to Orissa University of Agriculture and Technology (OUAT) laboratory, Bhubaneswar, Odisha, for different physicochemical analyses. The TDS, DO, Hardness (HCO_3_), TOC, and EC were measured as per the methods described by the American Public Health Association (APHA) (APHA, 2003). The different elemental composition of sediment was determined and quantified by inductively coupled plasma mass spectrometry (ICP-MS) technique. The DNA extraction was performed using the MoBio Power Soil DNA extraction kit following the manufacture’s instruction with some modifications to increase the yield and purity of the extracted DNA sample. First, the samples are heated at 65°C [instead of at room temperature (25°C)] in Power Bead tubes (included in the Power Soil kit) for 15 min, followed by vortexing for 5–8 min. The quality of the purified DNA was checked using 0.7% w/v agarose gel electrophoresis, and Nanodrop^TM^ 1,000 spectrophotometer (Thermo Scientific, Waltham, MA, United States) was used for quantification of DNA.

### Amplicon Library Preparation and Sequencing

The purified DNA was sent to Genotypic Technology Pvt. Ltd., Bangalore, for both library preparation and sequencing. Briefly, the 16S rDNA amplicon library of all four samples were prepared from the purified 50 ng DNA of each sample by PCR amplification of 423 bp fragment from V3-V4 hypervariable region using universal primer pairs i.e., 515F (5′-GTGCCAGCMGCCGCGGTAA) and 806R (5′-GGACTACHVGGGTWTCTAAT) (Alma et al., 2015; Apprill et al., 2015). The amplification and purification of amplicons were performed by using Platinum^TM^ PCR SuperMix High Fidelity (Thermo Fisher Scientific, Italy) and AMPure XP (Beckman Coulter, United States) kits (Rimoldi et al., 2018), respectively. Adapters and dual-index barcodes from Nextera XT Index Kit v2 were ligated to the target amplicons during second stage PCR followed by purification. All the four libraries were quantified using the Qubit dsDNA HS kit and the KAPA Library Quantification Kits (Kapa Biosystems Ltd., United Kingdom) on a Qubit 2.0 fluorometer (Life Technologies, United States) followed by normalization and pooling. The 2 × 250 bp paired-end sequencing of amplicon libraries was conducted on the Illumina MiSeq sequencer using the MiSeq reagent kit V2 (500 Cycle).

### Bioinformatics and Statistical Analysis

#### Analysis of Bacterial Community

For each sample, 16S miTAGs were extracted from the unassembled 16S rRNA gene sequence data using miTAGs_extraction_protocol (Logares et al., 2014) by HMMER v3.1 algorithms for both the forward and reverse sequences. For microbial diversity analysis, raw sequence reads and miTAGs extracted reads from all four samples (DJ01--DJ04) were uploaded to the MG-RAST v4.0.3 (Metagenome Rapid Annotation Subsystem Technology) server^1^ and processed according to their standard procedures. Briefly, the singled sequences (forward and reversed sequence) were paired using eight base pairs (bp) overlap settings and with a 10% maximum difference before being quality checked. SEED database in MG-RAST is subjected to a similarity search by its default quality control criteria with a maximum E-value of 10^–5^. DRISEE (duplicate read inferred sequencing error estimation) is used to remove the artificial replication reads and the Bowtie tool (Langmead, 2010) is used to screen out the near-exact matched sequence of *Homo sapiens* using SSU, RDP and Greengenes databases. The cd-hit was used to cluster at 97% identity and then the representative sequence reads of each clustered were picked using the BLAT^2^ algorithm with a minimum identity of 97%, 10^–5^ E-value and 50 bp minimum alignments. The highest number of BLAT hits was found against the SSU database compared to the other two databases (Greengene and RDP). Results from the SSU database were used for further downstream analysis. The bacterial root was classified at the domain level but not at other lower taxon levels, thus it represents the “unclassified”. Sequences that were not assigned to any bacterial root and were classified as “No Hits”.

### Prediction of Core OTUs

The core microbiome identifies the most common microbial member within a host population. Many studies have outlined that core microbiomes were observed using OTU detection in all samples or OTU detection in a certain percentage of samples, but this method is not reliable for core microbiome analysis (Hu et al., 2013). In the present study we implemented a multi-rank approach to selecting core OTUs, using the phyloseq^3^ R package. The Multi-rank approach is a statistical concept to identify numerically dominant taxa (Core-microbiota or Core-OTUs) in micro-biome analyses at different taxonomic levels i.e., Phylum, Class, Order, Family, Genus, and Species at a specific threshold (at cutoff 2) by implementing the taxonomic rank agglomeration^4^ through rank mean calculation (Elliot, 2014).

### Prediction of Functional Potential Using Tax4Fun and PICRUSt

The present work used the default settings of the PICRUSt (Phylogenetic Investigation of Communities by Reconstruction of Unobserved States) tool v1.1.4 (Langille et al., 2013) and Tax4fun (Aßhauer et al., 2015) to study the functional gene content in the Deulajhari samples based on the 16S rRNA amplicons. Representative sequences were aligned to reference sequences in the Ribosomal Database Project (RDP) classifier (Wang et al., 2007) and trained using the Greengenes (v13.8) database to execute the PICRUSt method. The Tax4Fun methodology, on the other hand, used the same sample sequence to allocate reference sequences in the SILVA SSU database (release 138). The input OTUs file for PICRUSt and Tax4Fun was generated through Quantitative Insight into Microbial Ecology (QIIME, v2020.11) (Caporaso et al., 2010) pipeline at a 97% sequence similarity. Tax4Fun converted the SILVA OTUs into KEGG (Kyoto Encyclopedia of Genes and Genomes) organisms, then normalized the results using the 16S rRNA copy number (found in NCBI genome annotations) (Aßhauer et al., 2015). However, to predict the KEGG function, Tax4Fun created a precomputed association matrix of KEGG Ortholog reference profiles to SILVA identified organisms by linearly merging the normalized taxonomic abundances into it. The Greengenes OTU dataset in BIOM format was used to predict metabolic functions by the PICRUSt approach. First, the OTU database was normalized by the known or expected 16S copy number abundance (Langille et al., 2013). Then, the PICRUSt script “predict_metagenomes.py” was used to generate predictive metagenomes from the generated Greengenes OTU dataset. In addition, Nearest Sequenced Taxon Index (NSTI) values were used to measure the accuracy of the PICRUSt predictions of the four samples (Langille et al., 2013; Staley et al., 2014).

### Carbohydrate Active Enzyme Prediction

To enrich the predicted functional profile obtained from Tax4Fun and PICRUSt, the genes and pathways with assigned with KO identifiers were catagorised within families and subfamilies of the Carbohydrate Active Enzyme (CAZyme) database^5^, to find the number and type of CAZyme functions present in the Deulajhari sample. The CAZYme database describes the families of structurally related catalytic and carbohydrate binding modules of enzymes that degrade, modify, or glycosidic bond formation. Finally, the pathway was reconstructed using the KEGG mapper.

### Statistical Analysis of Microbial Diversity and Functional Profiling

Estimation of the alpha diversity (Shannon, Sampson, and Chao) and UniFrac distance beta diversity indices among the samples were calculated and drawn using the microbiomeSeq R package (Ssekagiri et al., 2017). The number of common and unique OTUs at the genus level were visualized by plotting the Venn diagram^6^. A rarefaction curve was created to examine the species richness of all the organisms through the MG-RAST. Moreover, canonical correspondence analysis (CCA) triplot was constructed using PAST v4.03. The Pearson correlation was used to investigate the possible correlation between the top twenty genera with environmental parameters using the R software, and heatmap was plotted using the ggplot2. Only those OTUs with RA >0.05 are included for network analysis to reduce spurious correlation (Berry and Widder, 2014) and those OTUs had >0.5 positive associations correlation and <−0.5 negative associations correlation were included in the subsequent network analysis by Gephi v0.9.2. We used an additional low-abundance filter to exclude features whose relative abundance is >0.001 in any sample to explore the significant correlation of Mantel’s r statistics between the environmental parameter with taxonomic composition (miTAGs, MGRAST, and QIIME) and functional composition. Using this distance matrix, we calculated the calculated partial Mantel test’s correlation coefficient between compositional and environmental parameter distance (9,999 permutations) by ggcor and vegan package (Sunagawa et al., 2015). We constructed an evolutionary tree between representative OTUs at species level using phylogeny function in QIIME2 platform from the SILVA database at 1000 bootstrap value and visualized using Interactive Tree of Life (iTOL). Further statistical comparisons of the KEGG function proportions of hierarchical function (heatmap) among the samples were conducted in STAMP v2.1.3 (Parks et al., 2014). A web-based visualization application BURRITO (Browser Utility for Relating micRobiome Information on Taxonomy 72 and functiOn)^7^ was used to analyze the relationship between the microbial taxonomic and KEGG functional abundances across the sample.

## Results

### Site Description

Deulajhari spring complex, spread over 1.5 square kilometers, is surrounded by characteristic Pandanus plants of ∼7 meters in height. These plants provide a dense covering of around 18 live springs at a distance of about 10 meters. The elongated leaves (30 cm) shed from nearby plants form a 10–15 cm thick biomass bed and litter around all over the spring and on the water surface. Additionally, the sediments from the springs are captured by a concrete boundary wall of 2–3 feet height called a “kund” or in plural “kunda”, each of which is also known by a local nomenclature. We selected four sampling points targeting a gradient of temperature; the coldest had a temperature of 43°C-DJ01 (Himakunda), the moderate was 50°C-DJ02 (Labakusakunda), the hottest was 65°C-DJ03 (Taptakunda) and its outflow water was collected through a 20-meter man-made drain closer to the ground (0.4 meters height) with a 10°C lower temperature of 55°C-DJ04. Our earlier study examined a spring located ∼200 meters away from the present sampling location, at the entry point of the Deulajhari complex (Dixit et al., 2021) near the sanctum sanctorum of the Lord Shiva temple (Figure 1A). People travel from across the state to bathe in a man-made spring outflow of pond water there as part of religious customs, to cure themselves of several chronic ailments, and spring has become an important cultural pilgrimage site.

### Physiochemical Analysis of Four Spring Cluster Sample

The sediment of four samples did not show a difference in all physiochemical parameters through minor variation in pH from neutral to slight alkali (7.1 to 8.1), including TOC, sodium, chloride, zinc, and nitrate (Figure 1C). The quantity of EC (1253 μS/cm), sulfate (92 mg/L), and chloride (178 mg/L) were high in the sample from the high-temperature spring (65°C, DJ03). The low-temperature hot springs i.e., DJ01 showed the highest concentration of sodium (294 mg/L), fluorine (4 mg/L), phosphorous (5 mg/L), iron (0.18 mg/L), and zinc (0.2 mg/L), while DJ04 (moderated temperature, 55°C) had the highest concentration of TDS (545 mg/L), bicarbonate (230 mg/L), and TOC (3.76 mg/L). The chemical content such as zinc, iron, and calcium in the samples were detected as being quite similar in the moderate temperature sample (DJ02 and DJ04) (Figure 1C). However, the concentration of potassium and dissolving oxygen were similar in all samples.

### Sequence Generation

Out of 11.087 million reads, 85,71,16-18,41,176 were passed through quality control and GC content was found to vary at a range of 49.72–59.07% in all samples. Sequence reads were clustered into 1287 OTUs at 97% identity excluding singletons and were assigned to different microbial domains. The bacteria (95.96–96.61%) dominated, while the eukaryote (1.54–3.33%) and archaea (0.27–2.86%) contributed substantially less to the community (Table 1). 0.01–0.07% of sequences in each sample could not be identified.

**TABLE 1:** Statistics for 16s rRNA sequence.

**Category**	**DJ01**	**DJ02**	**DJ03**	**DJ04**
Total Sequence (bp)	235176960	219108693	216671286	437758281
No of HQ reads	936960	1706124	857116	1841176
Minimum/Maximum no. of reads	251	251	241–261	251
Percentages of HQ filter	52.14	51.16	75.01	93.74
Mean GC percent	49.72	58.51	59.07	58.49
Total OTUs Picked	995356	1560868	1273356	2076644
Bacteria	961690	1488893	1224708	1993030
Phylum	21	21	21	23
Class	48	48	47	53
Order	94	94	97	99
Family	166	185	164	180
Genus	300	341	288	320
Species	155	208	157	222

### Bacterial Taxonomic Diversity Analysis Between the Samples

**FIGURE 2:**
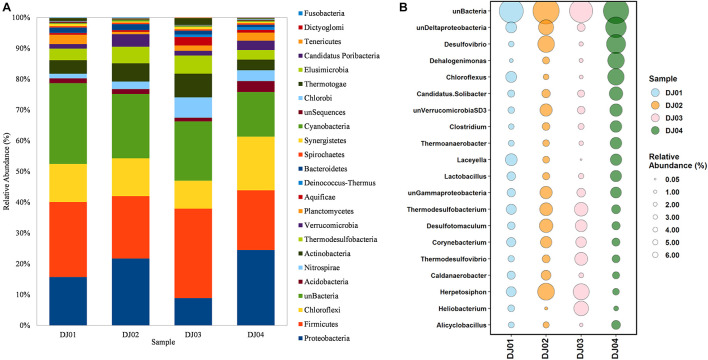
Comparisons of bacterial composition at phylum and genus level among the four sampling sites from Deulajhari thermal springs cluster. **(A)** The relative abundance percentage stacked bar graph at the phylum level. **(B)** The relative abundance of the top 20 bacterial phyla in the form of bubble plots. The size of the bubble indicates the relative abundances (%) of bacteria in each of the four sampling sites, and different color bubbles indicate different samples.

**FIGURE 3:**
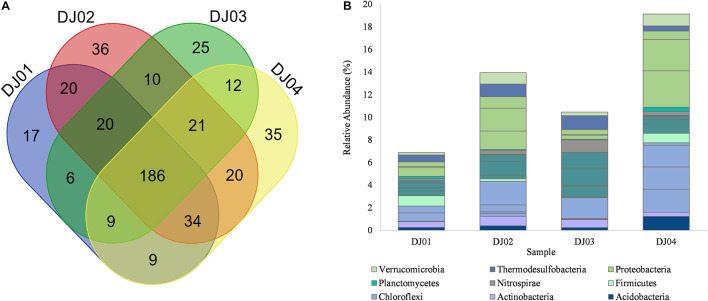
**(A)** Venn diagram representing the number of unique and common OTUs at the genus level. **(B)** Bar plot showing the relative abundance of core OTUs at the genus level identified by multi-rank mean approach in all samples of Deulajhari hot springs. Each genera is colored according to its respective phyla.

From the taxonomic information, the corresponding abundance of the species derived from all the samples was assigned. Amplicon sequences of Deulajhari hot springs were classified into 21 to 23 numbers of phyla, 44 to 53 class, 94 to 99 order, 166 to 185 family, 288 to 341 genus, and 155 to 222 species after filtering the OTUs (<10 among all samples). *Proteobacteria* was observed as the most dominant phyla in the DJ04 (8.62%) and DJ02 (5.71%) samples (Figure 2A). *Firmicutes* were detected to be the most abundant phyla in DJ03 (6.27%) and DJ01 (4.14%), and the second most abundant were DJ04 (6.82%) and DJ02 (5.32%), followed by *Chloroflexi*, *Actinobacteria*, and *Thermodesulfobacteria* (Supplementary Table 1A). *Deltaproteobacteria* was the most prominent class under the phylum *Proteobacteria* in DJ04 and DJ02 samples, and the order *Desulfovibrionales* being the most abundant. Similarly, *Clostridia* was the most abundant class in the DJ03 (5.41%) and DJ01 (2.21%) sample under *Firmicutes* phylum, with the order *Clostridiales* being the most abundant in all samples. Interestingly, all of the samples had a diversified bacterial community distribution at the class level (Supplementary Table 1B). A higher percentage of unclassified OTUs were observed in the DJ04 sample at the order level. *Herpetosiphonaceae* was the most prominent families in DJ02 (2.06%) and DJ03 (1.82%) sample whereas *Thermoactinomycetaceae* and *Desulfovibrionaceae* were the most predominant family in the DJ01 (0.89%) and DJ04 (2.74%) samples respectively (Supplementary Table 1C). At the genus level, DJ04 was dominated by *Desulfovibrio* (relative abundance 2.64%) from the family *Desulfovibrionaceae* (Supplementary Table 1D). The abundant genera in all samples were *Herpetosiphon*, *Lactobacillus*, *Chloroflexus*, *Oscillochloris*, *Deltaproteobacteria* (Unclassified), and *Thermodesulfobacterium*. The top twenty genera with their relative abundance were shown in a bubble plot (Figure 2B). Approximately 186 OTUs at the genus level were found to be common among the four samples, and 17, 36, 25, and 35 were uniquely present in DJ01, DJ02, DJ03, and DJ04, respectively, as shown in Figure 3A. The dominant bacteria in DJ04 (3.22%) and DJ01 (0.76%) was uncultured *Deltaproteobacteria*, whereas the dominant bacteria found in DJ02 (2.07%) and DJ03 (1.83%) *was Herpetosiphon aurantiacus.* All samples showed a high abundance of unclassified species (4.14 to 5.05%) along with uncultured *Deltaproteobacteria*, *Desulfovibrio alaskensis*, *Dehalococcoides ethenogenes*, and *Candidatus Solibacter usitatus* (Supplementary Table 1E).

To determine the core community among the springs cluster, we distinguished the “core” OTUs from a microbial community, rejecting the rare microbes within a body site in all samples. The multi-rank mean approach in the phyloseq R package provided nineteen “core” genera among all the samples. Despite the differences associated with temperature and pH, the core microbiome genera from all the samples are mainly composed of *Firmicutes* and *Chloroflexi* phylum. Around 68% of the “core” genera belong to *Firmicutes* (6 genera*: Laceyella*, *Lactobacillus*, *Desulfotomaculum*, *Caldanaerobacter*, *Heliobacterium*, and unclassified *Thermoanaerobacterales*), *Chloroflexi* (4 genera: *Dehalogenimonas*, *Chloroflexus*, *Dehalococcoides*, and *Herpetosiphon*) *Proteobacteria* (3 genera: unclassified *Deltaproteobacteria*, *Desulfovibrio*, unclassified *Gammaproteobacteria*), and *Acidobacteria* (1 genera: *Candidatus Solibacter*) (Supplementary Table 2). Moreover, the most dominant “core” OTUs at the genus level is unclassified *Deltaproteobacteria* (3.22%) and *Desulfovibrio* (2.75%) were observed in the DJ04 sample (Figure 3B). The complete details of all nineteen “core” genera are presented in Supplementary Table 2.

### Variation in Diversity Between the Samples

Rarefaction analysis revealed higher species richness in DJ02 and DJ03 as compared to DJ01 and DJ04 (Supplementary Figure 1). On the other hand, the Shannon diversity index indicated the highest bacterial diversity was in DJ02 which was followed by DJ04, DJ01 and DJ02. Estimates from Simpson’s and Peelou’s diversity indices suggest the bacterial diversity was nearly alike in all four sites. However, LCBD and community dispersal capacity correlated negatively with each other (Table 2).

**TABLE 2:** Diversity analysis of predicted bacterial communities.

**Sample name**	**Shannon**	**Simpson**	**Pielou’s evenness**	**Richness**	**LCBD**
DJ01	3.83	0.92	0.53	1251.00	0.23
DJ02	3.90	0.94	0.54	1131.30	0.14
DJ03	3.74	0.94	0.52	1156.52	0.31
DJ04	3.87	0.95	0.54	1038.09	0.32

### Correlation Between Microbial Community and Physicochemical Variables

**FIGURE 4:**
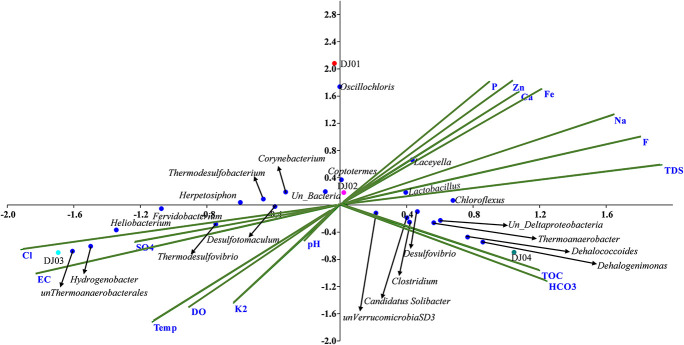
Canonical Correspondence Analysis (CCA) triplot between 16 physio-chemical features and relative abundance of genera (RA > 0.01% in at least two samples) across the samples. The blue dots represent the genera while the green line represents the individual physicochemical parameters, and samples were represented in different colored dots.

**FIGURE 5:**
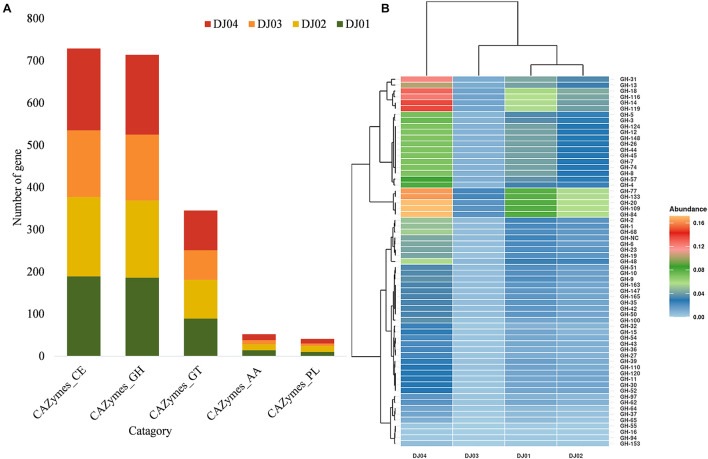
Distribution of predicted families and sub-families of Carbohydrate-Active Enzymes (CAZymes). **(A)** Bar plot showing the distribution of the total carbohydrate esterases (CE), glycoside hydrolase (GH), glycosyltransferase (GT), auxiliary activities (AA), and polysaccharide lyases (PL) families among the samples. **(B)** Heatmap with hierarchical clustering showing the distribution of different GH sub-families. Color shades indicate the relative abundance of the respective GH subfamily.

**FIGURE 6:**
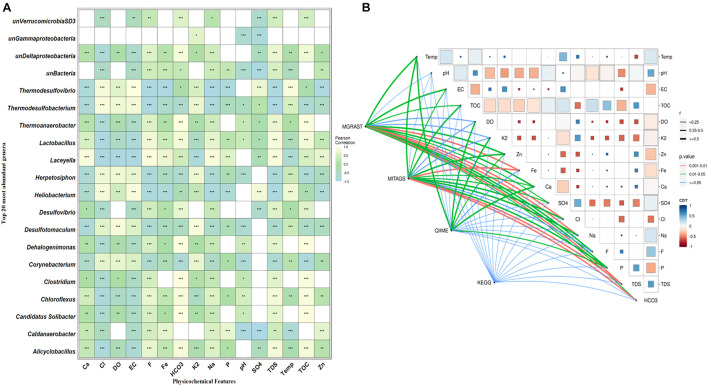
Pairwise statistical correlation between the environmental parameter and taxonomic community. **(A)** Pearson correlation between environmental factors and relative abundance of the top 20 of genera. Where *p < 0.05, **p < 0.01 and ***p < 0.01 indicates significant correlation. The horizontal rows represent the environmental parameter and the vertical rows represent the microbial community. Positive correlation showed light yellow color and negative correlation represented in sky blue color. **(B)** Mantel’s R statistical correlation between matrices of Pearson’s pairwise correlation among physicochemical parameters, taxonomic abundance obtained from three different methods (MG-RAST, miTags, and QIIME2), and functional profile (based on KEGG modules). In Pearson’s matrix, the color grade denoting the coefficient *r*-value between two factors. Similarly, the thickness of the edges represents the distance correlation value while their different color represents the statistical significance based on *p*-value calculated at 9999 permutations.

Canonical correlation analysis (CCA) and Pearson correlation tests were used to quantify the correlation between the environmental parameters and microbial communities of all samples (Figures 4, 5A). A total of 16 physiochemical variables (Figure 1C) were associated with microbial community structure. The environmental parameters such as HCO_3_, TOC, F, TDS, Fe, Cl, EC, and SO_4_ affected the microbial community structure (Figure 4). The genus, *Alicyclobacillus*, and *Laceyella* have shown a highly positive correlation with Ca, Fe, Na, F, and TDS (*p* < 0.001), and a negative correlation with K_2_, EC, and SO_4_ (*p* < 0.001). Among the predominant populations, *Candidatus Solibacter, Chloroflexus, Clostridium, Dehalogenimonas, Lactobacillus, Thermoanaerobacter*, and unclassified *Deltaproteobacteria* had a strong positive correlation with HCO_3_ and TOC (*p* < 0.001). However, *Heliobacterium* and *Thermodesulfovibrio* showed a positive correlation with temperature, SO_4_, Cl, EC, and a significant negative correlation with P, F, TDS, Ca, Fe, and Na (Figure 6A). The genus *Caldanaerobacter* and *Corynebacterium* showed a significant negative correlation with pH (*p* < 0.001), and no strong positive correlation was observed between the pH and microbial community structure. We observed that most of the environmental parameters had a non-significant correlation with unclassified *Verrucomicrobia* SD3 and unclassified *Gammaproteobacteria*. Similar type of correlation was observed from pearson’s correlation and mantel test. In contrast, it was observed that the HCO_3_, TDS, and TOC showed a significant positive correlation (*p* < 0.05) with the whole bacterial community composition (Figure 6B). No significant correlation was found for pH due to minor variation in pH values between the samples (Figures 6A,B).

### Prediction of Bacterial Functional Profiles

Functional microbiota profiles in the hot springs sediment samples were predicted based on individual OTUs using both Tax4fun and PICRUSt software, which revealed 427 and 451 groups at level 3 KEGG Orthology, respectively (Supplementary Tables 3A,B) and 25 functional categories at COG classification. At level 2, membrane transport, signal transduction, cellular community-prokaryotes, carbohydrate metabolism, and energy metabolism, etc., genes were among the major abundant functional pathways in all the samples. However, we observed that the abundance of signal transduction pathways was high in the DJ03 (33.75%) sample, and the abundance of membrane transport and carbohydrate metabolism were significantly increased in DJ04. A heat map of the top 50 abundant functional pathways in both software at level 2 has been presented in Supplementary Figures 2A,B. The details of the functional classification of the KEGG categories are shown in Supplementary Table 3. Furthermore, Burrito was used to predict the KEGG level’s carbohydrate metabolism, which showed a relationship among all the genera (Supplementary Figure 3). However, a very week correlation was observed between the environmental parameter and the predicted function (KEGG) by Mantel test (Figure 6B), as the prediction was performed using amplicon sequencing. The COG function distribution depicted the predominance of the functions related to cell wall/membrane biogenesis, Lipid transport and metabolism, carbohydrate transport metabolism, and metabolism in the amplicon data of all the samples, suggesting its functionality among all the hot springs (Supplementary Figure 4). Surprisingly, we did not find any significant difference between the samples in COG categories at the abundance level. However, the highest number of abundances was in translation (category J) in sample DJ04. An exclusive list of all predicted COG functions with their relative abundance can be found in Supplementary Table 4. Considering the vital importance of biomass-degrading activity in the hot springs due to the anticipated hydrocarbon contamination from the leaf litter bed, we focused our research on the responses of Carbohydrate Active Enzymes (CAZymes), targeting detection of more-efficient biomass-degrading enzymes.

### Identification of the Carbohydrate Genes Related to the Carbohydrate Degradation Pathway

Even though most of the KEGG functions were related to signal transduction and membrane transport (Supplementary Figures 2A,B), our study confirmed that many OTUs that matched the KEGG database (20–40%) originated from carbohydrate metabolism pathways. The total predicted function in the four samples was compared to the entries in the CAZymes database to identify enzymes and genes involved in the carbohydrate modification, biosynthesis, or breakdown. A high relative abundance (∼38–39.5%) of CAZymes belonging to the glycosyl hydrolase (GH) and carbohydrate ester (CE) families were predicted in all samples, but the percentage of Glycoside Transferase (GTs) (∼17–18%), Auxiliary Activities (AAs) (∼2.5–2.8%), and Polysaccharide Lyases (PLs) (∼1.5–2.8%) enzymes were lower (Figure 5A).

Glycosyl hydrolase (GHs) represented 38.72, 38.28, 39.5, and 38.64% of the CAZymes identified in DJ01, DJ02, DJ03, and DJ04 samples, respectively. Seven hypothetical families of GHs (GH1, GH4, GH13, GH14, GH47, GH57, and GH68) were identified exclusively in all samples (Figure 5B), and are involved in starch and sucrose metabolism. In total, 42 varied genes (out of 84 genes) and 23 different KO were identified in this pathway (Supplementary Figure 5A). Most of the Trehalose biosynthesis enzymes were predicted in hot springs samples. However, 10 enzymes were predicted in galactose metabolism pathways (Supplementary Figure 5B). Out of these, four enzymes were predicted in the generation of D-galactose and D-glucose, the starting substance being galactinol. In addition to other GH families such as GH3, GH5, GH,10, GH18, GH20, and GH84 were found to be associated with nucleotide sugar and amino sugar metabolism. The GT family was identified in all samples, among which the most abundant were the GT1, GT4, GT28, GT35, and GT51 subfamilies. The GT4 was responsible for many activities such as N-acetylcysteine deacetylase, nicotinamidase, indole acetamide hydrolase, etc. A higher abundance of GT families was found in DJ04 samples, and a low abundance was observed in DJ03 samples. Twenty-five percentages of carbohydrate esterase (CE) are shown in all samples (DJ01, DJ02, and DJ04) except DJ03 samples, which showed only 21% CE subfamilies. Genes of CE4 and CE8 families were present in all samples. The Lipopolysaccharide biosynthesis proteins were exclusively present in the DJ04 sample. The abundance of all the pathways was significantly higher in DJ04 samples. Details of the common and unique shared GH and CE families in all the samples are shown in Supplementary Table 5.

### Co-occurrence Network and Phylogenetic Analysis

**FIGURE 7:**
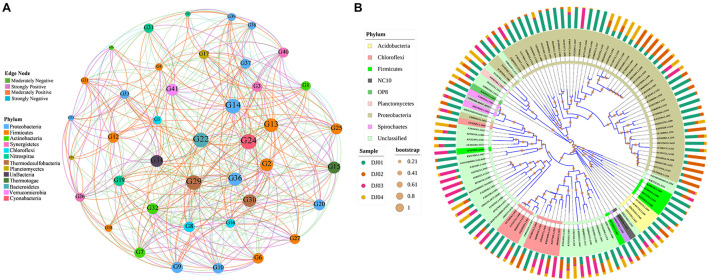
Network and clustering analysis of phyla. **(A)** Co-occurrence network analysis of the top 45 genera (moderate to strong Pearson’s correlation value). The edge thickness denotes the Pearson rank correlation value. Node denotes the genus code and is colored according to their respective phyla. The size of the node indicates the relative abundance percentages of the bacterial genus. The Colored of the node represents the strength (positive or negative) of correlation between the respective pairs of genera. **(B)** Mid-point rooted phylogenetic tree at genera level created using interactive Tree of Life (iTOL). The group of genera are colored according to their respective phylum. The outer bars graph indicates the relative abundance of respective OTUs and the bootstrap value of each node is represented by an orange-colored circle.

Co-occurrence network analysis was performed using Pearson correlation methods to characterize and visualize the co-occurrence patterns among the taxa of bacterial communities in our samples. In our results, the taxonomic network of the samples formed 41 nodes and 437 edges (Figure 7A). Only the genera with correlation values between 0.9 to 0.5 and −0.9 to −0.5 were plotted. The correlation network graph showed that the genus of *Firmicutes* exhibited a strong positive correlation with the genus of *Proteobacteria*, *Chloroflexi*, and *Nitrospirae* but a negative correlation with the *Actinomadura* of *Actinobacteria* phyla. Overall, we observed that few of the genera belonging to different phylum found together with strong positive correlation such as *Actinomadura* (phyla: *Actinobacteria*), unclassified *Epsilonproteobacteria* (phyla: *Proteobacteria*), *Caldanaerobacter* (phyla: *Firmicutes*), unclassified *Verrucomicrobia* SD3 (phyla: *Verrucomicrobia*). Finally, we highlighted the high level of co-occurrence between some genus of *Actinobacteria*, *Proteobacteria*, *Chloroflexi*, and *Firmicutes* (Figure 7A; *p*-value < 0.05). A detailed table with the genera code is presented in Supplementary Table 6. The phylogenetic analysis (Figure 7B) showed that OTUs could be grouped into nine clusters, and therefore, the analyzed sequences of the amplicon library are heterogeneous. It was found that the OTUs with the same phylum got clustered but interestingly, it was also found that some OTUs with different phyla such as *Acidobacteria* and *Firmicutes* were clustered together, whereas *Chloroflexi*, *Spirochetes*, and *Proteobacteria* showed another cluster. We highlighted the OTUs of the *Proteobacteria* phylum showing the highest number of coverage compared to others (Figure 7B).

## Discussion

### Geographical Location of the Spring Cluster

Deulajhari is located close to NE–SW trending shear zone on the southeast coast of India. Satellite image data locates Deulajhari on the southern fault of the Gondwana basin aligned to the Mahanadi lineament (Zimik et al., 2017). The Pandanus plant leaf litter thickly covers the space around the springs and some spring water surfaces amidst the dense forest. The leaves are long narrow blades with the characteristic aroma of Indian Kewda, which are known for their volatile essential oil content. Most previous studies on hot springs in Odisha are concerned with the geological or hydro-geochemical features (Pradhan and Jena, 2016; Zimik et al., 2017; Mahala, 2019). However, the real geological implications behind the genesis of diverse multiple geothermal manifestations occurring at a single location within a few hundred square meters have yet to be elucidated.

### Geo-Physiochemical and Microbial Features Determine the Genesis

The genesis of the thermal springs can be decrypted from the chemical signature of water it retains over time. However, these are influenced by the contamination of different water sources (connate, meteoric, and juvenile) and/or by seawater intrusion through deep faults near the coast (Gokgoz and Akdagoglu, 2016; Chatterjee et al., 2017; Zimik et al., 2017). Na and Cl dominance in Deulajhari springs compared to HCO_3_ and SO_4_ classify the springs as Chloride type/Na-Cl type, confirming their water to be mature and fast ascending (Zimik et al., 2017). The higher concentration of Na and Cl of this spring could be due to interaction with granite terrain while circulating through various structural lineaments and shears (Chandrasekharam and Antu, 1995; Nayak et al., 1998). A moderate amount of TDS also rules out the influence of seawater on water chemistry (Gemici and Filiz, 2001).

The higher concentration of HCO_3_ and TOC in DJ04 (Figure 1C) as well as their strong correlation with the bacterial community as depicted in the CCA plot (Figure 4) and Pearson analysis (Figure 6B) explains the possible mixing of runoff water from the leaf biomass litter. This leaf biomass has growth-promoting effects, shaping the microbial communities, with a significant cellulose degrader content (Bhandari and Nayal, 2007; Wan et al., 2015; López-Rebollar et al., 2021). When compared, the microbial diversity is found to be relatively higher in DJ04, than in DJ02, even though both are moderate temperature springs, probably owing to the higher organic matter content, and higher TDS, TOC, and HCO_3_ in DJ04 (Chan et al., 2017). This detection of a slight variation in physiochemical parameters might be due to contamination by subsurface water, prominently in DJ04, which is very close to the ground, and in DJ03 this could be due to very high temperatures. The altered physiochemical features due to high temperature are also reported in previous findings (Uribe-Lorío et al., 2019).

The lack of significant variation in alpha diversity indices (Shannon, Simpson, Pielou’s index) in all four springs exhibits uniformity to a greater extent and variation at a lower scale in their microbial community structure at higher taxon level (Table 2). These findings of overall similarity in diversity indices among four springs speak about the minor role of the geochemical features of water on the diversity of these spring points. However, the larger extent of homogeneity exhibited by these springs in geophysical, geochemical facts, and bacterial diversity at a higher taxonomic level establish the possibility of some interconnectivity among these thermal manifestations through plumbing systems deep under the earth’s crust.

The chemistry of their water with variable geothermal and geochemical environments is due to the possible influence and mixing up of deep heated water with underground shallow cold water, reservoir temperature, and rock-water interactions and provides clues about the ultimate origin of these varied thermal surface manifestations. These structural features are often interconnected, though remotely, with the resultant thermal activities of deep-seated intrusions and other heat-producing factors along with them (Mahala, 2019). There exists a difference in chemistry among these diverse thermal manifestations despite their close proximity occupying the same space, as also indicated by studies of one such world’s most active and explored hydrothermal complex at the Yellowstone National Park (YNP), United States (Gibson and Hinman, 2013). Furthermore, the overlapping and mixing of these individual hydrothermal activities are possible through the unique complex plumbing system underlying the earth’s crust, resulting in such variations.

### Correlation Between Microbial Community and Physicochemical Variables

While examining the community structure of individual springs through beta diversity analysis, we found that the springs with a moderate temperature (DJ02) of 50°C and (DJ04) 55°C have unique OTUs, of 36 and 35 respectively (Figure 3A). Moderate temperatures have been reported to harbor diverse microbiome (Badhai et al., 2015; Bennett et al., 2020). Minor deviation from the above trend, observed in the Pearson correlation between the environmental parameter and the microbial abundance genera (Figure 6A), could be due to the geochemical features playing an interfering role in individual springs (Li and Park, 2020). However, it was observed that the abundance of bacteria was higher in DJ04 samples (outflow) at a temperature 55°C. The possible reason could be the physical mixing of carbohydrate-contaminated shallow water percolating through leaf litter, chemical reaction, and unique biological activity at the outflow water.

High TOC and HCO_3_ due to the decomposition of plant leaf litter might promote the growth of specific bacteria (*Thermoanaerobacter*, *Desulfovibrio*, *Candidatus Solibacter*, *Dehalogenation*) at moderate temperatures (DJ04 55°C), as shown in Figure 6A (Edgar et al., 2011). This ultimately helps nutrient and carbon cycling in ecosystems and the release of dissolved organic matter back to the earth (Kindler et al., 2011). The differential location of phylotypes in different sectors in the CCA plot and Pearson heatmap supports the idea that environmentally relevant factors other than temperature also contribute to bacterial divergence in these hot springs (Klatt et al., 2013; Badhai et al., 2015; Sahoo et al., 2017; Kumar and Sharma, 2019). A significant positive correlation was depicted in Pearson heatmap and CCA analysis between sulfur content and sulfur reducing bacteria such as *Heliobacterium*, *Thermodesulfovibrio*, *Thermodesulfobacterium*, and *Herpetosiphon* (Lobo et al., 2012; Lavoie et al., 2020) by using them as electron acceptors to sustain several activities such as respiration, conserving energy and growth, in the absence of oxygen (Florentino et al., 2016).

The correlation network analysis suggested that the degree of correlation at the different taxa levels may not always depend on abundance. This suggests that interactions between microbes in any ecological niche may be independent of their abundance, as biogeochemical factors prevailing in the spring samples might significantly shape the community structure. One such report advocates a similar finding where *Chloroflexi* exhibited the highest positive correlation with other members instead of their lower abundance (Janssen, 2006; Narsing Rao et al., 2021). However, to establish the intensity of interaction, the habitat affinities, and shared physiologies among the inhabiting members need to be investigated (Proulx et al., 2005; Janssen, 2006; Barberan et al., 2012).

### Temperature Favors Selective Photosynthetic Moderate Thermophiles

*Chloroflexi* and *Cyanobacteria* are the most common photosynthetic dwellers of moderately thermophilic slightly alkaline hot springs. However, while *Cyanobacteria* acts as the primary producer, the *Chloroflexi* utilizes the metabolites as their carbon source (Badhai et al., 2015; Chan et al., 2017). In our study, we found a non-linear trend in their abundance across a gradient of temperature from 43 to 65°C in four springs at neutral to slightly alkaline pH, though the highest abundance was found in moderately heated spring samples DJ04 (55°C) and DJ02 (50°C). Furthermore, a differential richness of genus was observed at different temperature profiles at the phylum level. Temperature regulates their abundance differently and favors specific taxa of the phylum and, thus, their composition in a hot spring is known to influence their activity and number (Bennett et al., 2020). Higher abundance of two major green biofilm communities forming phyla *Chloroflexi* and *Proteobacteria* in open-to-sky slightly alkaline spring outflow at DJ04 (pH 8.1) is due to moderate temperature (55°C), high concentration of sulfur (87 mg/l) and sufficient amount of sunlight, as well, contamination of percolated hydrocarbon (3.76 mg/l, TOC) from the bed of fallen leaf biomass litter onto its water surface from the dense population of pandanus tree surrounding the spring (Lee et al., 2018). This needs further characterization to establish their biofilm-producing ability.

### Hydrocarbon Contamination Drives Enrichment of Spring CAZome

Valorization of biomass through microbial transformation employs novel thermophilic CAZymes to produce food, feed, and fuel for their robust adaptability. Our previous study undertook a non-cultivable analysis of the entry point of the Deulajhari spring detected several biomass degrading bacteria *Pseudomonas*, *Aeromonas*, *Chryseobacterium sp.*, and *Raoultella*, as this is the established approach for discovering evolved CAZomes (Dixit et al., 2021). Based upon this earlier study, we explored the present sample locations DJ01-04 (around 300 meters away from the previous), which are surrounded by a thick mat of Pandanus leaf litter, anticipating a higher abundance of CAZymes and a higher gradient of TOC. The involvement of 400–500 putative genes as well as detection of a majority (∼80%) of CAZymes belonging to the GH family 3 and GH family 5, which are predominantly responsible for breakdown, biosynthesis, and modification of carbohydrates, indicate the higher leaf litter decomposing potential of Deulajhari spring water cluster (Nyyssonen et al., 2013). The detection of 72 genes that are precisely responsible for the metabolism and absorption process of starch and sucrose through the production of α-amylase, α-glucosidases, glucoamylase, and isoamylase abundantly in all the spring waters is supported by previous genome studies of *Colwellia* and *Rheinheimera* (Methe et al., 2005). The highest diversity and abundance of almost all hydrocarbon family genes and pathways in CAZomes of DJ04 indicate the highest possible carbohydrate metabolism. Biomass-degrading genes and enzymes, due to mixing with spring water, were also reported in foliage contaminated outflow of the Malaysian Y-shaped Sungai Klah hot spring (Lee et al., 2018). The hottest spring DJ03, bounded by 2–3 feet high rectangular man-made masonry, which provides structural protection above the ground, was least exposed to leaf fall and hydrocarbon contamination and thus, exhibited the lowest abundance of genes (*n* = 400) for CAZymes. The abiotic stress of hydrocarbon in the spring might have forced the microbial community to catabolize energy-rich stored carbohydrates such as cellulose, starch, and glycogen, etc., into simpler forms such as glucose as a source of carbon and energy to drive different metabolic processes (Demoling et al., 2007).

### Survivability Strategies of True Thermophiles at High Temperature

Microorganisms exhibiting optimal growth and multiplication at a temperature above 60°C are considered true thermophiles. Their relentless adaptation to survival physiology and ability to thrive under extreme conditions leads to genomic evolution (Xian et al., 2018). The resultant abundance of genes necessary for survival strategies was predicted to be functional in high-temperature spring sample DJ03 (65°C). DNA repair and replication genes and pathways are the adaptive features of microorganisms responsible for taking care of DNA damage due to extreme temperature (Xian et al., 2018). These thermophiles are highly evolved and thrive against the environment’s deleterious effects compared to mesophiles (Li et al., 2014; Saxena et al., 2016). A high abundance of translational machinery directs a higher percentage of A + G in mRNA, making the thermophiles more stable and protects them from adverse environments (Xian et al., 2018). The dominance of energy metabolism pathways in the 65°C spring sample is another tactic followed by the thermophiles used to counter the heat stress as a physiological response, which is further supported by previous reports of the upregulation of Glycolytic pathway-related proteins *Geobacillus* sp. (Xian et al., 2018). Despite the adapted survivability strategies of the dwellers of spring DJ03, it shows low species richness, which indicates that a smaller portion of the microbiome acclimatizes in these extreme conditions.

### Core and Unique Members of the Spring Cluster

The core microbiota is a crucial component of any ecological niche involved in prevailing biological activity and exhibits a strong association with it irrespective of the temperature and other environmental conditions (Shade and Handelsman, 2012; Parfrey et al., 2018). The exclusive presence of few chemolithoautotrophic bacterial genus *Desulfonauticus* (*Proteobacteria*) and *Gramella* (*Bacteroidetes)* in DJ01 only could be due to the prevailing moderately low temperature and their suitability to inhabit the prevailing bacteria (Hetzer et al., 2008; Mayilraj et al., 2009; Mandal et al., 2019). Whereas the detection of a good number of thermophiles in DJ03 could be because of higher temperature, sulfur, and iron content (Nunoura et al., 2008; Davis-Belmar and Norris, 2009). However, 36 genera were found solely in DJ04 samples at 55°C in slightly alkaline pH, mostly belonging to *Proteobacteria* and *Actinobacteria* phyla. Of these, moderate thermophilic genus *Marichromatium* (Sucharita et al., 2010) and highly efficient bioethanol producing *Zymomonas* were found abundantly in DJ04 (Jones-Burrage et al., 2019). The stability of the core OTUs is due to their strong associations with springs, despite the occurrence of a significant shift in microbiota. The majority of core bacterial genus were possibly involved in the biogeochemical recycling of minerals and biodegradation of present hydrocarbons in spring samples (Yoshikawa et al., 2016; Yang et al., 2018). Therefore, these core microbes may play an important role in regulating the metabolic functions and maintaining the overall community stability of the thermal springs.

## Conclusion

The present study undertook a comprehensive examination of the microbial communities of India’s unique hot spring complex at Deulajhari, which has multiple outflows. The taxonomical analysis revealed that the microbial community was dominated by bacteria in which *Proteobacteria* is the most dominant phyla in DJ04 and DJ02. *Firmicutes* were detected as the most abundant phyla in the DJ03 and DJ01 geothermal sites. Several bacterial members correlate with the TOC and sulfur that predicted the possible functioning of the carbon and sulfur cycle. A large number of hydrocarbon family genes and pathways in CAZomes were predicted in all the locations, with the highest in DJ04, forecasting active biomass-degrading pathways in this sample. Taken together, the results derived from this study highlight the importance of the genomic potential of the Deulajhari hot springs. Future research should be directed at characterizing the identification of bacterial members through *in vitro* studies. In addition, shotgun sequencing and transcriptome analysis need to be employed to reveal the actual metabolic potential of the hot spring microbial communities.

## Data Availability Statement

The data analyzed in this study can be found in online repositories. The link to the repository along with accession number(s) is as follows: https://www.ncbi.nlm.nih.gov/sra/SRX2470965; https://www.ncbi.nlm.nih.gov/sra/SRX2470962; https://www.ncbi.nlm.nih.gov/sra/SRX2470963; and https://www.ncbi.nlm.nih.gov/sra/SRX2470961.

## Author Contributions

SaD: conceptualization, data curation, formal analysis, investigation, methodology, visualization, writing – original draft, and review and editing. MG: conceptualization, data curation, formal analysis, methodology, visualization, and writing-review and editing. ES: conceptualization, project administration, resources, supervision, writing-original draft, and review and editing. RS: investigation and writing – review and editing. SuD: writing – review and editing. LM: writing – review and editing. SM: writing – review and editing. NS: writing – review and editing. HA: Designed the hypothetical hot spring plumbing system. All authors contributed to the article and approved the submitted version.

## Conflict of Interest

The authors declare that the research was conducted in the absence of any commercial or financial relationships that could be construed as a potential conflict of interest.

## Publisher’s Note

All claims expressed in this article are solely those of the authors and do not necessarily represent those of their affiliated organizations, or those of the publisher, the editors and the reviewers. Any product that may be evaluated in this article, or claim that may be made by its manufacturer, is not guaranteed or endorsed by the publisher.

## Footnotes

1
http://metagenomics.anl.gov/

2
http://www.genome.org/cgi/doi/10.1101/gr.229202

3
http://joey711.github.io/phyloseq/

4
https://github.com/microDM/core-microbiome

5
http://www.cazy.org/

6
http://bioinformatics.psb.ugent.be/webtools/Venn

7
http://borensteinlab.com/software_burrito.html

## Supplementary Material

Supplementary Figure 1 Rarefaction analysis of all four metagenomes from Deulajhari. All the samples are rarefied at the same no of reads. The average count of species found in each sample is plotted on the *y*-axis.

Supplementary Figure 2 The diversity of predicted profile of KEGG pathways. The functional gene content is predicted then mapped to pathways based on normalized 16S rRNA gene copy number. The diversity of the top 50 pathways predicted using **(A)** PICRUSt and **(B)** Tax4Fun. The pathways are clustered hierarchically according to the weightage of their relative abundance among all four samples.

Supplementary Figure 3 Interactive illustration (generated from BURRITO) of the relationship between the composition of the taxonomic and predicted functional profile among all four thermal springs. The differently abundant super-pathways of the metabolism category are highlighted. The carbohydrate metabolism super-pathway is linked to respective genera. The size of each node (circle) in the taxonomic and functional tree represents the average abundance of that entity. The width of linked edges to different genera represents the average share of the functional pathway.

Supplementary Figure 4 The bar plot shows the Clusters of Orthologous Groups (COG) predicted in all four samples. The relative abundance of each COG function is represented on the *x*-axis.

Supplementary Figure 5 Reconstructed **(A)** Starch and Sucrose metabolism and **(B)** Galactose Metabolism sub-pathway. The map was created by mapping predicted enzyme EC numbers against the reference pathway map through KEGG Mapper (https://www.genome.jp/kegg/mapper/). The enzyme EC numbers highlighted by the green box were predicted in the Deulajhari spring cluster.
