# Supplementary figures and images for: Bacterial Diversity and CAZyme Potential Revealed in Pandanus Rich Thermal Spring Cluster of India: A Non-cultivable 16S rRNA Sequencing Approach
Source: Front Microbiol. 2021 Nov 25;12:760573. doi: 10.3389/fmicb.2021.760573 (PMC8656282; doi:10.3389/fmicb.2021.760573)

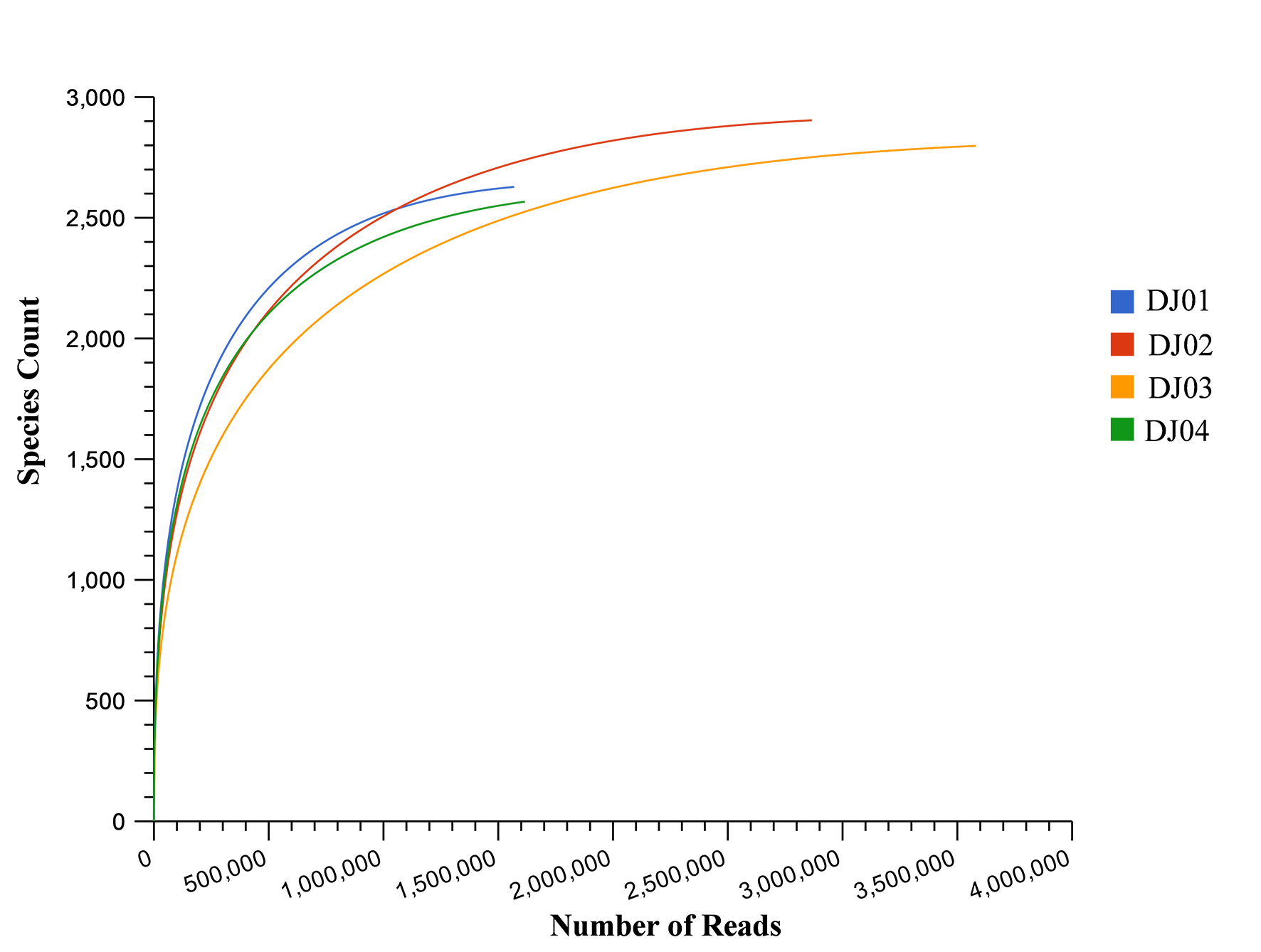

Supplement: Supplementary Figure 1 — Rarefaction analysis of all four metagenomes from Deulajhari. All the samples are rarefied at the same no of reads. The average count of species found in each sample is plotted on the y-axis. [file Image_1.TIF]

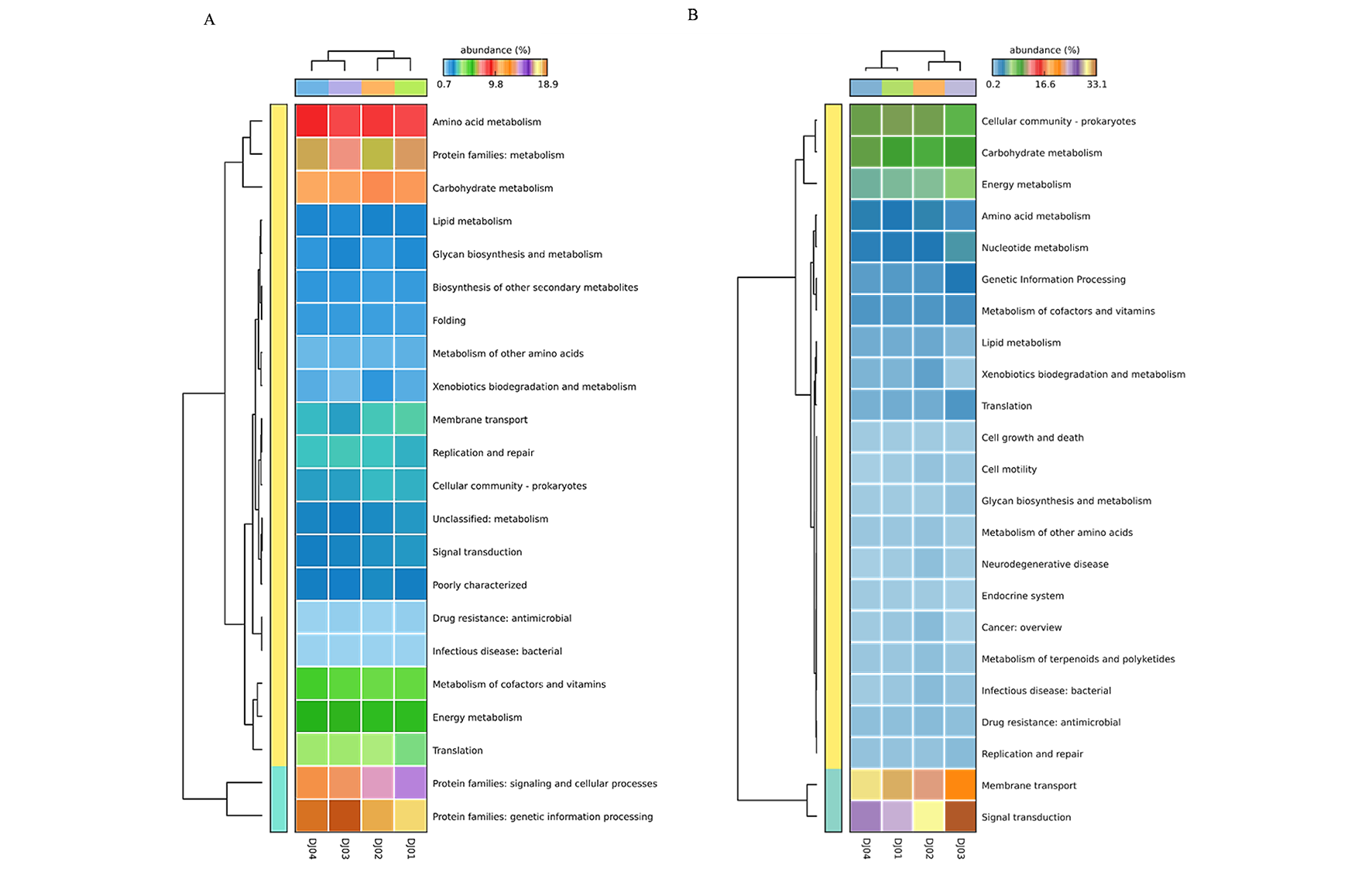

Supplement: Supplementary Figure 2 — The diversity of predicted profile of KEGG pathways. The functional gene content is predicted then mapped to pathways based on normalized 16S rRNA gene copy number. The diversity of the top 50 pathways predicted using (A) PICRUSt and (B) Tax4Fun. The pathways are clustered hierarchically according to the weightage of their relative abundance among all four samples. [file Image_2.TIF]

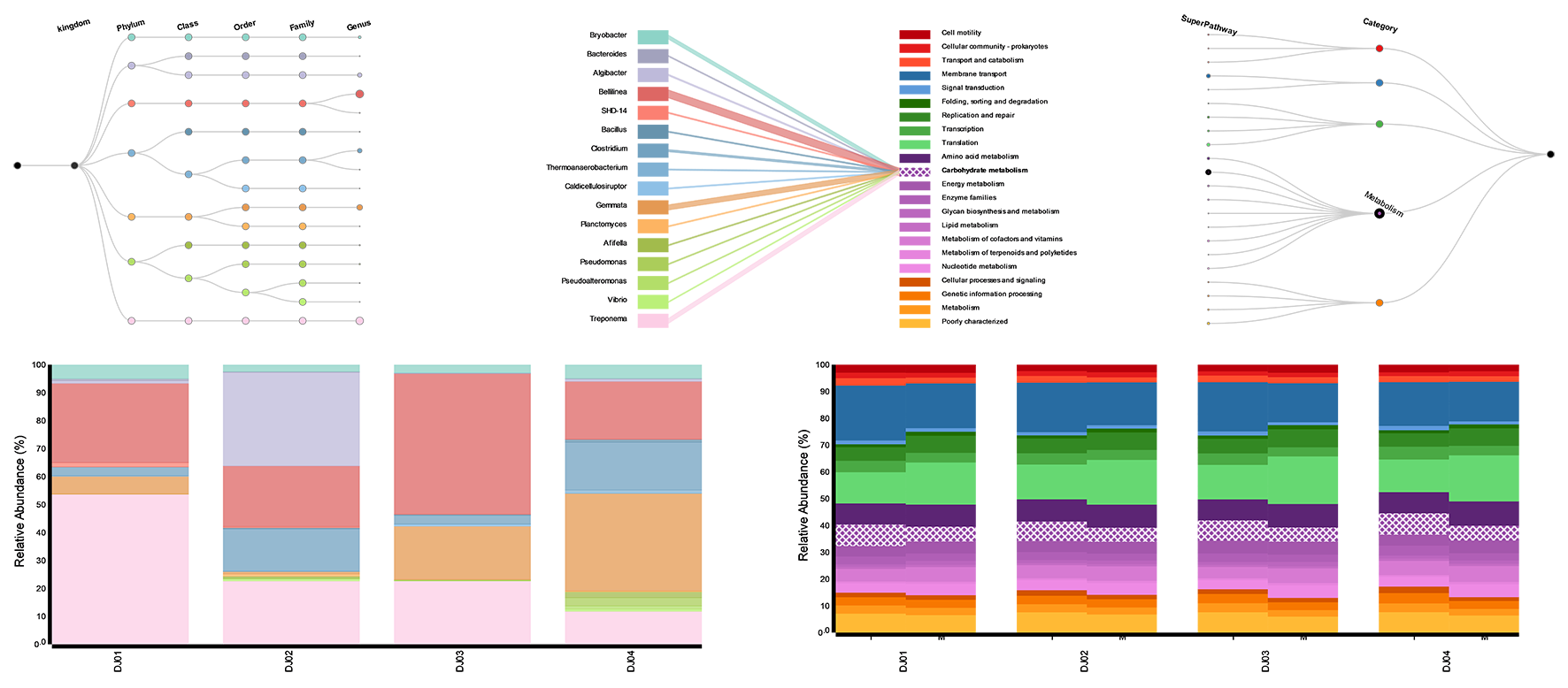

Supplement: Supplementary Figure 3 — Interactive illustration (generated from BURRITO) of the relationship between the composition of the taxonomic and predicted functional profile among all four thermal springs. The differently abundant super-pathways of the metabolism category are highlighted. The carbohydrate metabolism super-pathway is linked to respective genera. The size of each node (circle) in the taxonomic and functional tree represents the average abundance of that entity. The width of linked edges to different genera represents the average share of the functional pathway. [file Image_3.TIF]

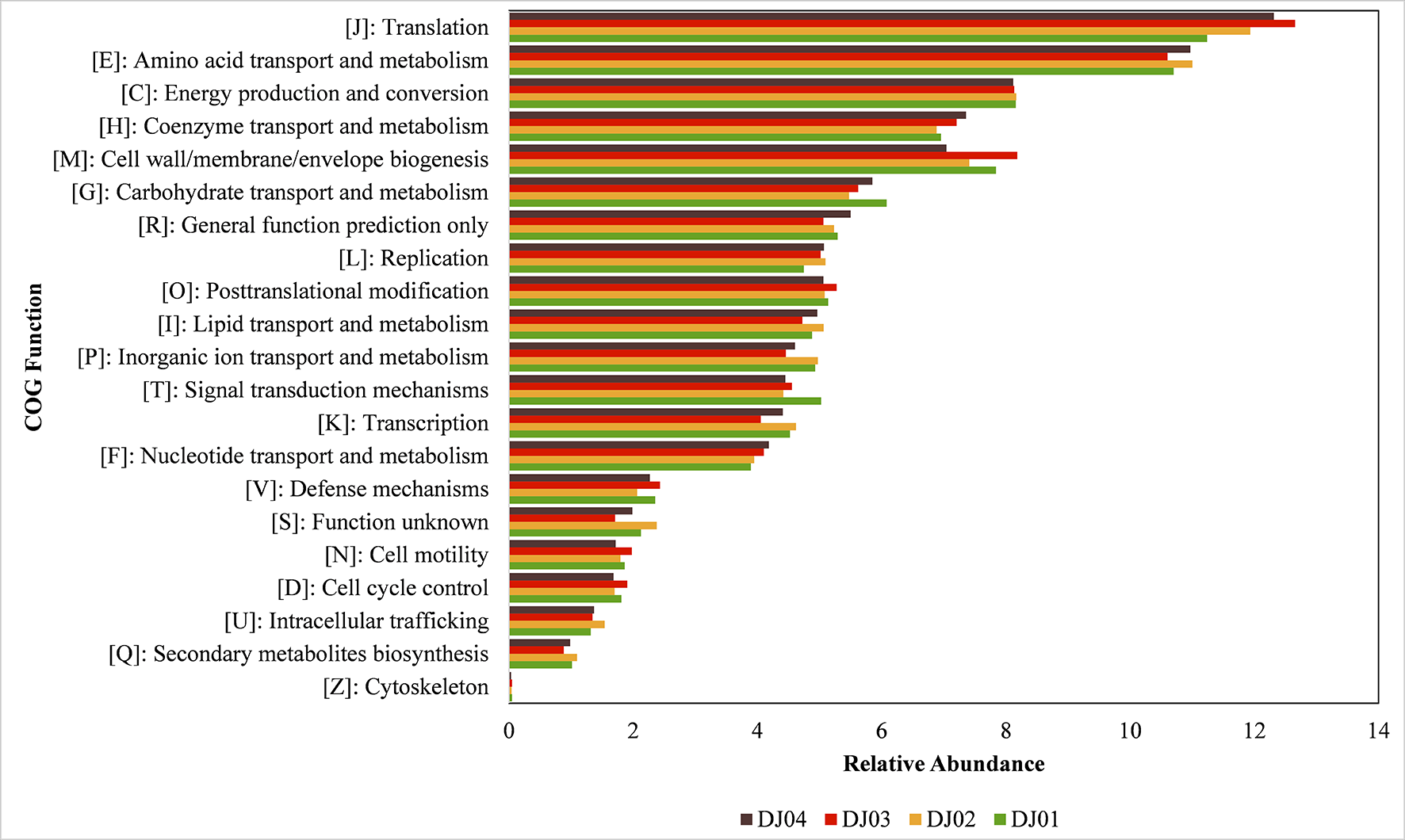

Supplement: Supplementary Figure 4 — The bar plot shows the Clusters of Orthologous Groups (COG) predicted in all four samples. The relative abundance of each COG function is represented on the x-axis. [file Image_4.TIF]

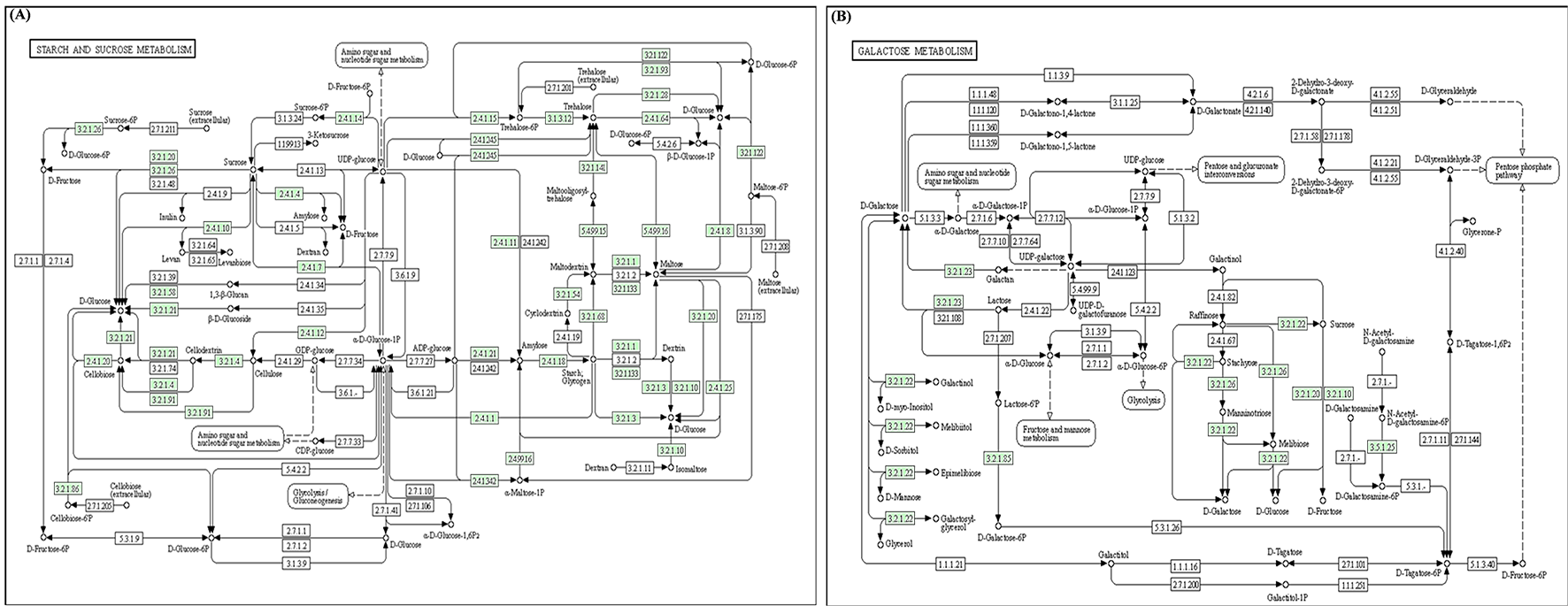

Supplement: Supplementary Figure 5 — Reconstructed (A) Starch and Sucrose metabolism and (B) Galactose Metabolism sub-pathway. The map was created by mapping predicted enzyme EC numbers against the reference pathway map through KEGG Mapper (https://www.genome.jp/kegg/mapper/). The enzyme EC numbers highlighted by the green box were predicted in the Deulajhari spring cluster. [file Image_5.TIF]
